# Comparison of interferon gamma release assay and *CXCL9* gene expression assay for the detection of *Mycobacterium bovis* infection in African lions (*Panthera leo*)

**DOI:** 10.3389/fcimb.2022.989209

**Published:** 2022-09-16

**Authors:** Rachiel Gumbo, Tashnica T. Sylvester, Sven D. C. Parsons, Peter E. Buss, Robin M. Warren, Paul D. van Helden, Michele A. Miller, Tanya J. Kerr

**Affiliations:** ^1^ DSI-NRF Centre of Excellence for Biomedical Tuberculosis Research, SAMRC Centre for Tuberculosis Research, Division of Molecular Biology and Human Genetics, Faculty of Medicine and Health Sciences, Stellenbosch University, Tygerberg, South Africa; ^2^ Afrivet Business Management, Newmark Estate Office Park, Pretoria, South Africa; ^3^ Veterinary Wildlife Services, Kruger National Park, Skukuza, South Africa

**Keywords:** African lion, *CXCL9*, gene expression assay, IFN-γ, interferon gamma release assay, *Mycobacterium bovis*

## Abstract

*Mycobacterium bovis* (*M. bovis*) infection has been identified in both domestic and wild animals and may threaten the conservation of vulnerable species including African lions (*Panthera leo*). There is a need to develop accurate ante-mortem tools for detection of *M. bovis* infection in African big cat populations for wildlife management and disease surveillance. The aim of this study was to compare the performances of two immunological assays, the QuantiFERON^®^-TB Gold Plus (QFT) Mabtech Cat interferon gamma release assay (IGRA) and QFT *CXCL9* gene expression assay (GEA), which have both shown diagnostic potential for *M. bovis* detection in African lions. Lion whole blood (n=47), stimulated using the QFT platform, was used for measuring antigen-specific *CXCL9* expression and IFN-γ production and to assign *M. bovis* infection status. A subset (n=12) of mycobacterial culture-confirmed *M. bovis* infected and uninfected African lions was used to compare the agreement between the immunological diagnostic assays. There was no statistical difference between the proportions of test positive African lions tested by the QFT Mabtech Cat IGRA compared to the QFT *CXCL9* GEA. There was also a moderate association between immunological diagnostic assays when numerical results were compared. The majority of lions had the same diagnostic outcome using the paired assays. Although the QFT Mabtech Cat IGRA provides a more standardized, commercially available, and cost-effective test compared to QFT *CXCL9* GEA, using both assays to categorize *M. bovis* infection status in lions will increase confidence in results.

## Introduction

Bovine tuberculosis (bTB) is a chronic infectious disease of animals caused by *Mycobacterium bovis* (*M. bovis*) and constitutes a zoonotic risk especially in developing countries (Ayele et al., 2004; Müller et al., 2013). *Mycobacterium bovis* infection has been identified in domestic animals, as well as a wide range of captive and free-ranging wildlife and may threaten the conservation of vulnerable species like the African lion (*Panthera leo*) (Lécu and Ball, 2011; Bernitz et al., 2021; O’Halloran et al., 2021). Lions, which are classified either as maintenance or spill over hosts, are frequently exposed to *M. bovis* in endemic areas because of predation on infected prey such as African buffaloes (*Syncerus caffer*) (de Lisle et al., 2002; Miller et al., 2015; Viljoen et al., 2015).

Because of the slow progression of disease, there are significant challenges in early detection of bTB in free-ranging wildlife, since most infected animals can shed viable mycobacteria before manifestation of visible clinical signs (Michel et al., 2006; Meiring et al., 2021). Therefore, to prevent the risk of introducing disease into new populations, movement of animals from endemic areas is restricted, unless there are accurate tests that can be used for screening. One of the greatest challenges for wildlife management is the lack of available diagnostic tools. The tuberculin skin test (TST), which measures the delayed-type hypersensitivity reaction to injected *M. bovis* purified protein derivative (PPD), is the only widely available ante-mortem test for large felids, but it requires capture and immobilization twice within three days, which increases mortality associated risks to both animals and staff (Keet et al., 2010; Miller et al., 2019).

Blood-based tests that only require a single capture are appealing for use in free-ranging wildlife. Frequently *in vitro* tests measuring cell-mediated immune (CMI) responses may detect infected animals before the onset of delayed-type hypersensitivity skin response (Pollock and Neill, 2002; Pollock et al., 2006). Although serological tests have been explored and shown diagnostic potential in lions, results suggest that antibody responses cannot regularly be detected during an early infection but only later after development of disease (Miller et al., 2012; Miller et al., 2019). Therefore, there has been a focus on CMI responses to mycobacterial antigens in African lions. Assays based on measuring interferon gamma (IFN-γ) concentrations in QuantiFERON^®^-TB Gold Plus (QFT) plasma and changes in chemokine (C-X-C motif ligand 9; *CXCL9*) gene expression have recently shown diagnostic potential as screening tests for *M. bovis* infection in African lions (Olivier et al., 2015; Gumbo et al., 2022). The aim of this study was to (a) screen African lions from *M. bovis* endemic and exposed populations for *M. bovis* infection using currently available previously validated assays (QFT Mabtech Cat IGRA and QFT *CXCL9* GEA) to identify immune sensitized individuals and (b) compare the agreement between GEA and IGRA results for detection of *M. bovis* sensitization in African lions.

## Methods

### Animal sampling and blood stimulation

Ante-mortem blood (n=47) and post-mortem tissue (n=12) samples for this study were selected from a larger cohort of opportunistically acquired free-ranging African lion samples which originated from known *M. bovis*-endemic (Kruger National Park and Hluhluwe-Imfolozi Park) and exposed (private game reserves) lion populations in South Africa. Wildlife populations were classified as exposed or endemic for *M. bovis*, based on previous history of *M. bovis* infection in any wildlife species in the area (Bernitz et al., 2021). Whole blood from each lion was collected in BD Vacutainer^®^ lithium heparin tubes (Becton, Dickinson and Company, Sparks, MD 21152, USA) after which 1 ml aliquots of heparinized whole blood from each animal was transferred to a set of QuantiFERON^®^-TB Gold Plus (QFT) tubes (Qiagen, Hilden, 40724, Germany), which included QFT nil tube (negative control), QFT TB2 specific antigen tube, and QFT mitogen tube (positive control). Pokeweed mitogen (PWM; 10 μg/ml final concentration in phosphate buffered saline (PBS); Sigma-Aldrich, St. Louis, MO 63103, USA) was added to the QFT mitogen tube to ensure adequate stimulation. Tubes were incubated at 37°C for 24 hours, after which blood was transferred to a new 2 ml microcentrifuge tube and centrifuged at 2000 x g for 15 minutes. Plasma supernatant was harvested and frozen at -80°C, while the remaining cell pellet was stabilised in 1.3 ml of RNALater^®^ (Ambion, Austin, TX 78744, USA) and stored at -80°C for downstream analyses. The *M. bovis* infection status of the subset of euthanized lions was confirmed using mycobacterial culture of post-mortem tissues (Kerr et al., 2022) followed by speciation of positive cultures using genomic regions of difference (RD) polymerase chain reaction (PCR) (Warren et al., 2006) and spoligotyping (Kamerbeek et al., 1997)

Immobilization of animals, blood collection, euthanasia, and tissue sampling were done by South African Veterinary Council–registered wildlife veterinarians for procedures unrelated to this study. Ethical approval for this study was granted by the Stellenbosch University Animal Care and Use Research Ethics Committee (Protocol SU-ACU-2017-1489) and the Stellenbosch University Biological and Environmental Safety (REC: BES) Research Ethics Committee (Protocol SU-BEE-2021-22561). Section 20 approval was granted by the South African Department of Agriculture, Land Reform and Rural Development (12/11/1/7/2A-1143NC, 12/11/1/7/2A-1181NC, 12/11/1/7/2A-1182C).

### QFT *CXCL9* gene expression assay

Tubes containing the cell pellet stabilized in RNALater^®^ were centrifuged at 15000 x g for 1 minute and the supernatant discarded, after which RNA extraction was performed using the RiboPure™-Blood Kit (Ambion), according to manufacturer’s instructions. The concentration (ng/µl) and quality (A260/A280 and A260/A230 ratios) of extracted RNA were measured using a Nanodrop 1000 spectrophotometer (ThermoFisher Scientific, Wilmington, NC 28401, USA). Using the QuantiTect^®^ Reverse Transcription Kit (Qiagen), cDNA was synthesized from extracted RNA, according to manufacturer’s instructions. An Applied Biosystems Veriti™ Thermal Cycler (ThermoFisher Scientific) was used for incubation during reverse transcription.

Using the cDNA, produced from QFT nil, TB2 antigen, and mitogen whole blood samples, real-time quantitative PCR (real-time qPCR) assays were performed in triplicate as described by Olivier et al. (2015). This assay has been previously optimized and validated for use in African lions (Olivier et al., 2015). For each animal, the abundance of *CXCL9* mRNA, measured in relation to reference gene tyrosine 3-monooxygenase/tryptophan 5-monooxygenase activation protein, zeta polypeptide **(** *YWHAZ*) for normalization, was calculated to determine immune activation (using QFT mitogen and nil samples) and immune sensitization (QFT TB2 antigen and nil samples) using the 2^-ΔΔCq^ method as previously described by Olivier et al. (2015). Presence or absence of *M. bovis* immune sensitization in each lion was assigned using an assay cut-off value of ≥ 5-fold change (Olivier et al., 2015).

### QFT Mabtech Cat IGRA

Plasma (QFT nil, TB2, and mitogen) samples for all African lions that were used in this study were screened in duplicate wells using the Mabtech Cat IFN-γ ELISA ^Basic^ kit (catalogue no. 3122-1H-20; Mabtech AB, Nacka Strand, SE-131 28, Sweden). The enzyme-linked immunosorbent assay (ELISA) protocol was performed following manufacturer’s instructions, using a 1:4 plasma dilution in ELISA diluent (PBS, 0.05% Tween^®^20, 0.1% bovine serum albumin). The mycobacterial antigen-specific IFN-γ concentrations were determined as described by Gumbo et al. (2022). The *M. bovis* sensitization status was assigned using an assay cut-off value of 33 pg/ml (Gumbo et al., 2022).

### Data analysis

For each animal, the relative abundance of target gene *CXCL9* mRNA was measured by first averaging the raw triplicate Cq values of nil, TB2 antigen, and mitogen samples for target and reference genes. The average nil Cq values were subtracted from either the average TB2 antigen or the average mitogen Cq values (ΔCq) before subtracting the reference ΔCq value from the target ΔCq value to obtain the ΔΔCq. Lastly, the assay result, which measures the relative fold change in abundance of the target gene (2^-ΔΔCq^), was calculated as described by Livak and Schmittgen (2001). To measure IFN-γ concentrations, for each lion, the true optical density (OD) results of a sample assayed in duplicate, was calculated by subtracting the mean OD of the negative control (background control) from the mean OD of samples for QFT nil, TB2, and mitogen prior to interpolating IFN-γ concentrations (pg/ml) using a cat recombinant IFN-γ standard curve. The proportions of positive and negative results, using assay-specific lion cut-off values, from paired samples tested using the QFT Mabtech Cat IGRA and QFT *CXCL9* GEA were compared using McNemar’s test (McNemar, 1947) with Yates correction (Yates, 1934) (https://www.graphpad.com/quickcalcs/mcNemar1/). Cohen’s kappa analysis was also used as another measure of agreement (McHugh, 2012) (https://www.graphpad.com/quickcalcs/kappa1/). The strength of the association between paired IGRA and GEA results was determined by the Spearman correlation coefficient (Schober et al., 2018) calculated using GraphPad Prism 7 for Windows (version 7.04, GraphPad Software, Inc., San Diego, CA 92108, USA; www.graphpad.com). Results of analyses were considered statistically significant if p-value < 0.05.

## Results

**Figure 1:**
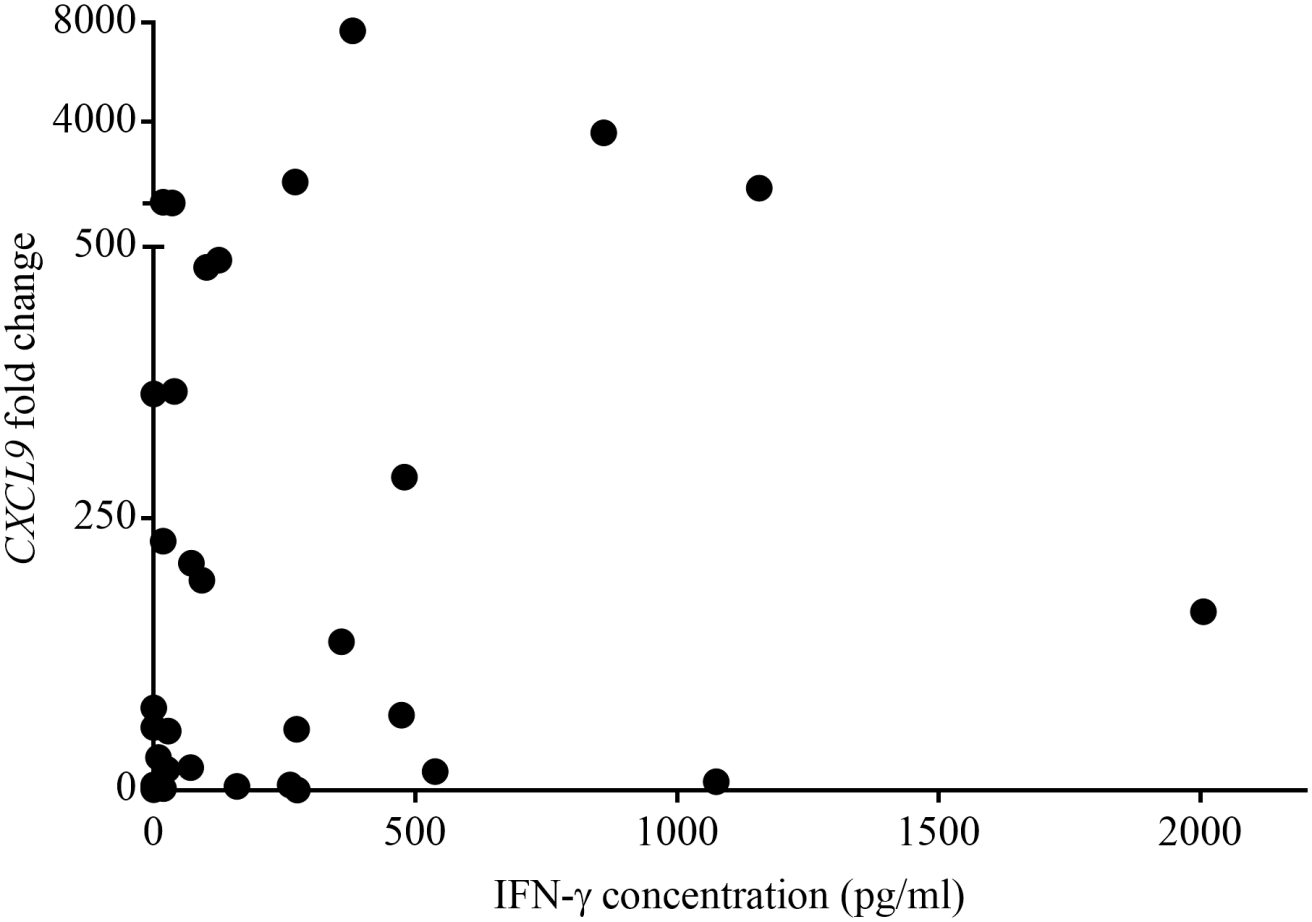
Paired numerical results of QuantiFERON^®^-TB Gold Plus (QFT) *CXCL9* gene expression assay (GEA) and QFT Mabtech Cat interferon gamma release assay (IGRA) for a cohort of *Mycobacterium bovis* exposed African lions. Spearman rank correlation showed a statistically significant moderate positive association between the results obtained using GEA and IGRA (ρ = 0.59, p < 0.0001).

**Table 1** shows the QFT *CXCL9* GEA and QFT Mabtech Cat IGRA results for all euthanized lions (n=12) in this study whose *M. bovis* infection status was confirmed using mycobacterial culture. The QFT *CXCL9* GEA was able to correctly identify all mycobacterial culture-confirmed infected and uninfected animals (**Table 1**). While the QFT Mabtech Cat IGRA was able to correctly recognize all mycobacterial culture-negative animals, it incorrectly classified one *M. bovis* infected lion (KNP 21/632) as uninfected (**Table 1**). Using the assay specific cut-off values to categorize lions as test positive or negative, the paired test results from a larger cohort of African lions were determined and are presented in **Table 2**. This larger cohort included the 12 mycobacterial culture-confirmed *M. bovis* infected and uninfected lions as well as 35 lions with unknown infection status. There was no statistical difference between the proportions of test positive African lions determined by the QFT Mabtech Cat IGRA as compared to the QFT *CXCL9* GEA (p = 0.15). There was also a moderate association when numerical results from the QFT Mabtech Cat IGRA and QFT *CXCL9* GEA were compared (ρ = 0.59; p < 0.0001; **Figure 1**) (κ = 0.50; 95% confidence interval (CI): 0.26 - 0.73).

**Table 1:** Results of mycobacterial culture confirmed *Mycobacterium bovis* infected and uninfected African lions using the QuantiFERON^®^-TB Gold Plus (QFT) Mabtech Cat interferon gamma release assay (IGRA) and QFT *CXCL9* gene expression assay (GEA). Test results were categorized as positive if the QFT *CXCL9* GEA value was ≥ 5-fold change and the QFT Mabtech Cat IGRA value was ≥ 33 pg/ml.

Lion ID	*Mycobacterium bovis* culture status	QFT *CXCL9* GEA		QFT Mabtech Cat IGRA	
		TB antigen specific fold change (2^-ΔΔcq^)	Test Result	TB antigen specific IFN-γ concentration (pg/ml)	Test Result
PPGR-Ple.1	Neg	0.39	Neg	0	Neg
PPGR-Ple.2	Neg	0.77	Neg	0	Neg
PPGR-Ple.3	Pos	17.15	Pos	538	Pos
PPGR-Ple.4	Pos	7.66	Pos	1075	Pos
PPGR-Ple.5	Neg	4.99	Neg	0	Neg
PPGR-Ple.11	Neg	1.49	Neg	0	Neg
ZRR-Ple.1	Pos	481.04	Pos	101	Pos
ZRR-Ple.2	Pos	136.55	Pos	359	Pos
KNP 17/612	Pos	68.91	Pos	474	Pos
KNP 19/372	Pos	7687.68	Pos	380	Pos
KNP 20/20	Pos	704.28	Pos	37	Pos
KNP 21/632	Pos	30.13	Pos	9	Neg

ID – lion identification number; QFT – QuantiFERON^®^-TB Gold Plus; GEA – gene expression assay; IFN-γ – interferon gamma concentration; *CXCL9* – chemokine (C-X-C motif) ligand 9; IGRA – interferon gamma release assay; Neg – negative; Pos – positive.

**Table 2:** Paired results of African lions tested using QuantiFERON^®^-TB Gold Plus (QFT) Mabtech Cat interferon gamma release assay (IGRA) and QFT *CXCL9* gene expression assay (GEA), based on species-specific assay cut-off values (33 pg/ml, ≥ 5-fold change, respectively) were compared using McNemar’s test (p = 0.15) and Cohen’s kappa analysis (κ = 0.50; 95% confidence interval (CI): 0.26 - 0.73).

	IGRA positive	IGRA negative	TOTAL
***CXCL9* GEA positive**	19	9	28
***CXCL9* GEA negative**	3	16	19
**TOTAL**	22	25	47

IGRA, interferon gamma release assay; GEA, gene expression assay; *CXCL9*, chemokine (C-X-C motif) ligand 9.

## Discussion

Although the development of specific immunological tests for diagnosis of bovine TB in wild felids has so far been relatively limited, measurement of mycobacterial antigen-specific IFN-γ production and expression of antigen-specific *CXCL9* mRNA have shown diagnostic potential for detecting *M. bovis* infection in African lions (Olivier et al., 2015; Gumbo et al., 2022). In this study, the performance of these two previously optimized assays for African lions were compared in order to improve detecting *M. bovis* sensitized individuals. The results of this study indicate that there was no significant difference in the probability of obtaining the same diagnostic result for *M. bovis* immune sensitization status when using either QFT Mabtech Cat IGRA or QFT *CXCL9* GEA with assay-specific lion cut-off values. This was supported by the moderate association values when numerical results were compared. Therefore, it appears that both assays are appropriate for ante-mortem detection of *M. bovis* infected lions.

Cytokine assays are widely used for TB diagnosis (Pollock et al., 2013; Bernitz et al., 2021). Antigen-specific IFN-γ is the most widely recognized biomarker of *M. bovis* infection in veterinary studies (Smith et al., 2021b) as well as for *M. tuberculosis* infection in people (Klautau et al., 2018; Goletti et al., 2022). Studies in humans and cattle have also shown that *CXCL9*, also known as monokine induced by gamma interferon (MIG), has potential value as a biomarker for TB diagnosis and monitoring (Chung et al., 2015; Kumar et al., 2019; Palmer et al., 2020). A previous study has also shown that in lions, there was greater upregulation of *CXCL9* than either *IFN-γ* or *CXCL10* (Olivier et al., 2015). During *M. bovis* infection, IFN-γ promotes cell-mediated immune responses by activating macrophages and granuloma formation (Silva Miranda et al., 2012; Palmer et al., 2022). Additionally, IFN-γ, which is essential for the expression of *CXCL9* and *CXCL10* by macrophages and several cell types during the recruitment of T-cells at the site of infection, is produced to enhance host protection (Algood et al., 2003; Cooper et al., 2011). The genes *CXCL9* and *CXCL10* code for monokine-induced by gamma interferon (MIG) and IFNγ-induced protein 10 (IP-10), respectively, which have shown diagnostic potential for TB in both humans and animals (Smith et al., 2021b). Therefore, it is not surprising that *CXCL9* expression correlates with enhanced T-cell IFN-γ production induced by TB-specific antigens ESAT-6/CFP-10 in this study. The study by Olivier et al. (2015) highlighted the increase in mean Cq values between TB antigen-stimulated samples and unstimulated samples, which were 8.8 for *CXCL9*, 5.6 for *CXCL10*, and 1.0 for *IFN-γ*. Although *CXCL10* was upregulated, *CXCL9* had the greatest upregulation of the three genes evaluated (*CXCL9, CXCL10, IFN-γ*) in *M. bovis* infected African lions, showing its potential as a diagnostic marker. A mean Cq difference of 1 is not sufficient to differentiate between unstimulated and stimulated sample, hence *IFN-γ* GEA was not suitable, although an antigen-specific IGRA has been shown to be useful for the detection of *M. bovis* in African lions (Gumbo et al., 2022). Although the *CXCL9* GEA and IGRA results were associated in the current study, there was a single discordant result (lion KNP 21/632) for a culture-confirmed positive lion. Since both assays have the same reported imperfect sensitivity (87.5%) with high specificity (100%) (Olivier et al., 2015; Gumbo et al., 2022), this was not unexpected. Therefore, findings from this study support use of either IGRA or *CXCL9* GEA for detection of *M. bovis* infection in lions.

Since both assays require blood samples, use of QFT IGRA or *CXCL9* GEA provides an advantage over the TST, which necessitates immobilization of lions twice in a 72-hour period (Keet et al., 2010; Miller et al., 2019), while the blood-based assays can be completed within 36-48 hours of blood collection. In addition, blood-based assays have a significantly reduced turnaround time compared to mycobacterial culture which can take weeks for results (Hines et al., 2006). These assays also employ mycobacteria-specific peptides for stimulation compared to PPDs used in the TST, which would decrease the likelihood of false positive results due to cross-reactivity to nontuberculous mycobacteria (Keet et al., 2010; Viljoen et al., 2019). In addition to the convenience of using a blood-based assay, the QFT stimulation platform allows generation of samples that can be tested in both immunological assays. Archived samples then provide material for repeated testing, retrospective studies, or the development of new assays (Chileshe et al., 2019; Smith et al., 2021a). Therefore, the QFT stimulation platform offers a valuable addition to the toolbox for evaluating antigen-specific immunological responses in African lions.

The majority of lions had the same diagnostic outcome using the paired assays, although only a moderate association in numerical results was detected. This could be explained by the influence of IFN-γ as a key cytokine in cell-mediated immune responses in TB, and more specifically, its role in promoting expression of *CXCL9* (Lande et al., 2003; Metzemaekers et al., 2018). An explanation for the moderate association is that this study compared cytokine protein production versus gene expression assays. Complex protein formation and post-transcriptional splicing, and translational regulation might result in a lag in detecting cytokine proteins compared to mRNA (Guo et al., 2008). Hence the 24-hour incubation period may not have been optimal for both assays simultaneously, therefore, future studies should consider exploring QFT incubation times appropriate for African lions. Discordant results might also result from technical errors or differences in sample handling of the cell pellet and plasma (Guo et al., 2008).

Although cytokine mRNA expression and protein production are important and complementary TB diagnostic tools in veterinary and human studies, choosing a single approach for testing wild felids is challenging. One limitation of cytokine GEA is that it can be laborious in terms of RNA isolation and reverse transcription. In addition, it requires greater technical skill, specialized laboratory equipment, and more expensive reagents, which may not be available, especially if testing wildlife. The QFT Mabtech Cat IGRA uses commercially available antibodies, making it technically easier, more standardized, and cost-effective (approximately $64/animal) than the QFT *CXCL9* GEA (approximately $158/animal), especially when screening large numbers of African lions. However, when feasible, the use of both assays may increase confidence in categorizing the *M. bovis* infection status in lions (Sivakumaran et al., 2021). Overall, more research is needed to focus on screening and investigating additional TB biomarkers, such as MIG, in felids by extrapolating from humans and other species. Recommendations for future studies include evaluation of additional cytokine release assays such as interferon gamma-induced protein 10 (IP-10), MIG, tumour necrosis factor alpha (TNF-α), interleukin-2 (IL-2) used in biosignatures in human TB, as well as circulating serum biomarkers that could be incorporated into a diagnostic algorithm for felids using a convenient set of samples (Yong et al., 2019; Morris et al., 2021). The current study was able to utilize a single antigen-stimulated blood sample for both the IGRA and *CXCL9* GEA, which facilitates analyses, especially when using a convenient stimulation platform such as the QFT. Assays for TB detection in wildlife require that samples be easily collected, processed and transported from remote field conditions. Therefore, this study highlights how novel application of tools for disease screening can be used in conservation programmes for wild felids in southern Africa, as well as managed populations globally.

## Data availability statement

The original contributions presented in the study are included in the article/supplementary materials. Further inquiries can be directed to the corresponding author.

## Ethics statement

Ethical approval for this study was granted by the Stellenbosch University Animal Care and Use Research Ethics Committee (Protocol SU-ACU-2017-1489) and the Stellenbosch University Biological and Environmental Safety (REC: BES) Research Ethics Committee (Protocol SU-BEE- 2021-22561).

## Author contributions

The work presented here was carried out in collaboration between all authors. RG, MM, and TJK developed and designed the study. RG and TS conducted experiments and analysed the data. PB was involved with sample collection and clinical data. Original manuscript draft was prepared by RG and SP contributed to data analysis. MM, RW, and PH provided funding for the study. All authors reviewed the manuscript and approved the published version of the manuscript.

## Funding

Financial support for this research was provided by the South African Medical Research Council (SAMRC) Centre for Tuberculosis Research, South African Veterinary Foundation (SAVF), and the National Research Foundation (NRF) South African Research Chair Initiative (SARChI grant 86949). Funding to RG was provided through a Stellenbosch University (SU) Postgraduate Scholarship (2021) and German Academic Exchange Service (DAAD) In-Region Scholarship SUN MBHG South Africa (2022). Funding to TK was provided through DSI-NRF PDP Fellowship within the SAMRC Centre for Tuberculosis Research. Opinions expressed, and conclusions arrived at, are those of the authors and are not necessarily to be attributed to the funders.

## Acknowledgments

The authors acknowledge Guy Hausler, Tebogo Manamela, Leana Freese, and Dr. Lufuno Netshitavhadulu from South African National Parks (SANParks) Veterinary Wildlife Services, Kruger National Park (KNP); Lin-Mari de Klerk-Lorist and Louis van Schalkwyk from Skukuza State Veterinary Office; Alicia McCall from Hluhluwe State Veterinary Office as well as Dr. M. Toft, K. Odendaal and S. Naylor from private game reserves (PGR) in KwaZulu-Natal (KZN), South Africa for their contribution to this research.

## Conflict of interest

The authors declare that the research was conducted in the absence of any commercial or financial relationships that could be construed as a potential conflict of interest.

## Publisher’s note

All claims expressed in this article are solely those of the authors and do not necessarily represent those of their affiliated organizations, or those of the publisher, the editors and the reviewers. Any product that may be evaluated in this article, or claim that may be made by its manufacturer, is not guaranteed or endorsed by the publisher.
